# The Topobiology of Chemical Elements in Seabird Feathers

**DOI:** 10.1038/s41598-017-01878-y

**Published:** 2017-05-17

**Authors:** Nicholas R. Howell, Jennifer L. Lavers, Sayaka Uematsu, David Paterson, Daryl L. Howard, Kathryn Spiers, Martin D. de Jonge, Tracey Hanley, Richard Garrett, Richard B. Banati

**Affiliations:** 1 Australian Nuclear Science and Technology Organisation (ANSTO), Lucas Heights, Australia; 2 Institute for Marine & Antarctic Studies, University of Tasmania, Tasmania, Australia; 3 James Cook University, Cairns, Australia; 4 NRDA Asia, Tokyo, Japan; 5 Australian Synchrotron, Melbourne, Australia; 6 National Imaging Facility at Brain and Mind Centre (BMC), Faculty of Health Sciences, University of Sydney, Sydney, Australia

## Abstract

The highly organized morphogenesis of bird feathers holds important phylo- and ontogenetic information on the evolution of birds, organogenesis, tissue regeneration, and the health status of individual animals. Altered topobiological patterns are regularly used as retrospective evidence for disturbed developmental trajectories due to the past exposure to environmental stressors. Using the most advanced high-resolution (5–70 µm) X-ray fluorescence microscopy (XFM), we describe in the feathers from three species of Procellariiformes hitherto unknown, depositions of elements (Zn, Ca, Br, Cu, Fe) that are independent of pigmentation or any underlying variation in density or polymer structure. In the case of Zn, the pattern across several species of Procellariiformes, but not other species, consisted of highly regular bands of Zn numbering 30–32, which may reflect the estimated number of days of active feather growth or the duration of the moult period. Thus, speculatively, the highly consistent Zn pattern might be the result of a so far unknown diurnal systemic regulation rather than local heterogeneity amongst the follicular stem cells.

## Introduction

The intricate structural, cellular and molecular patterns observed during the growth of bird feathers provide important insight into the phylogenesis of birds, as well as the fundamental biological processes that govern organogenesis and regeneration. Anomalies in the expected orderly patterns, such as fault bars, are often used as a retrospective record of the exposure to environmental stressors and thus health status of the animal^1, 2^.

**Figure 1:**
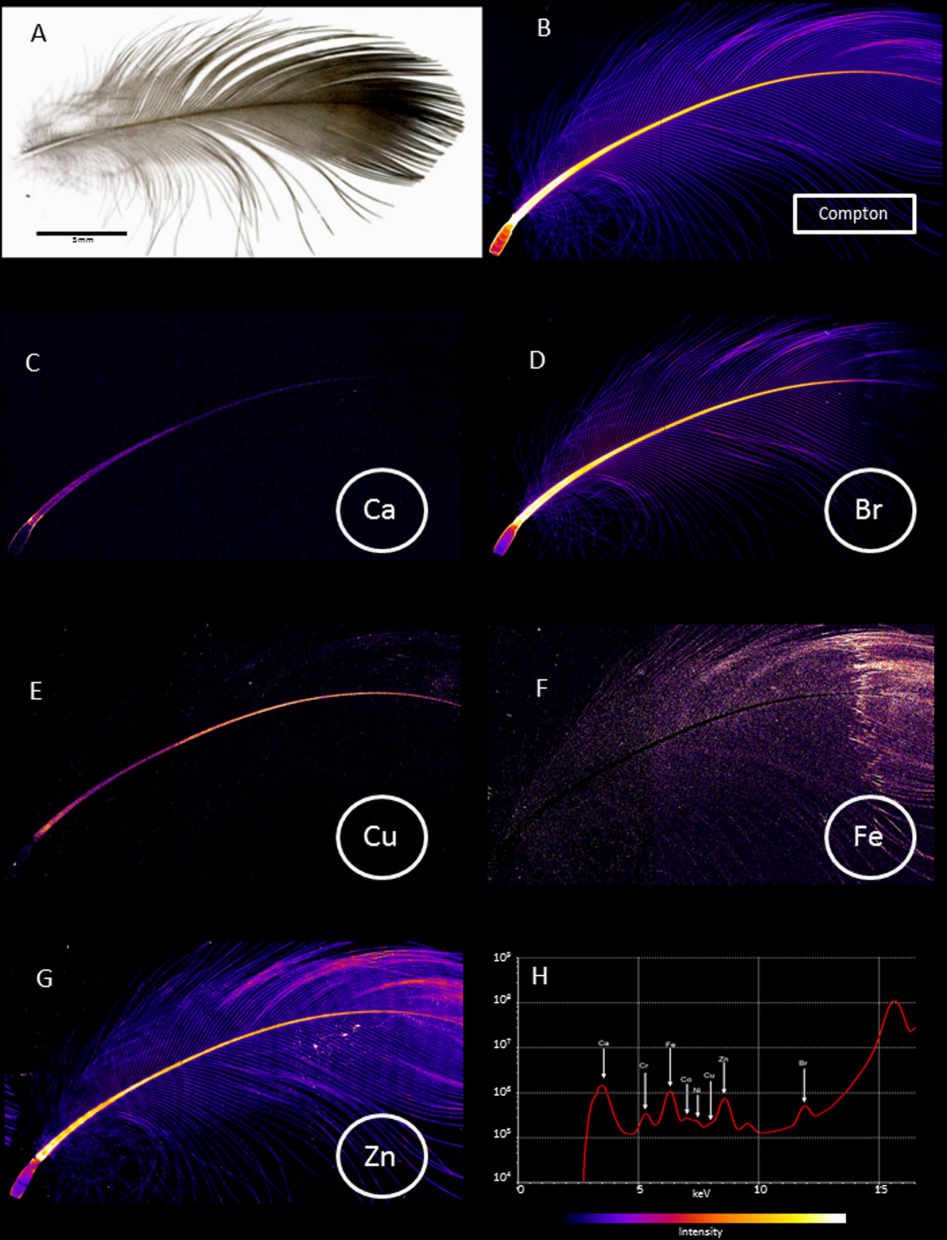
High resolution X-ray fluorescence microscopy images of elemental distributions within a breast feather from a Flesh-footed Shearwater (*Ardenna carneipes*). (**A**) Photographic image of a sample feather; (**B**) reconstructed Compton inelastic scatter; (**C**) Ca distribution; (**D**) Br distribution; (**E**) Cu distribution; (**F**) Fe distribution; (**G**) Zn distribution; (**H**) typical X-ray fluorescence spectrum obtained from Flesh-footed Shearwater breast feathers. The maximum values for the observed elements are; Ca, 7650 ppm; Cu, 113 ppm; Zn, 488 ppm; Br, 279 ppm; Fe, 345 ppm.

Here, we report so far unseen spatial distribution maps of unprecedented resolution and sensitivity for the atomic elements Zn, Ca, Br, Cu and Fe (Fig. 1) in single breast and wing feathers of three species of Procellariiformes, the Flesh-footed (*Ardenna carneipes*), Streaked (*Calonectris leucomelas*) and Short Tailed (*Ardenna tenuirostris*) Shearwaters, and, for comparison, a number of other species of various phylogenetic distance, using the X-ray Fluorescence Microprobe (XFM) and the Maia Spectroscopy Detector System at the Australian Synchrotron.

**Figure 2:**
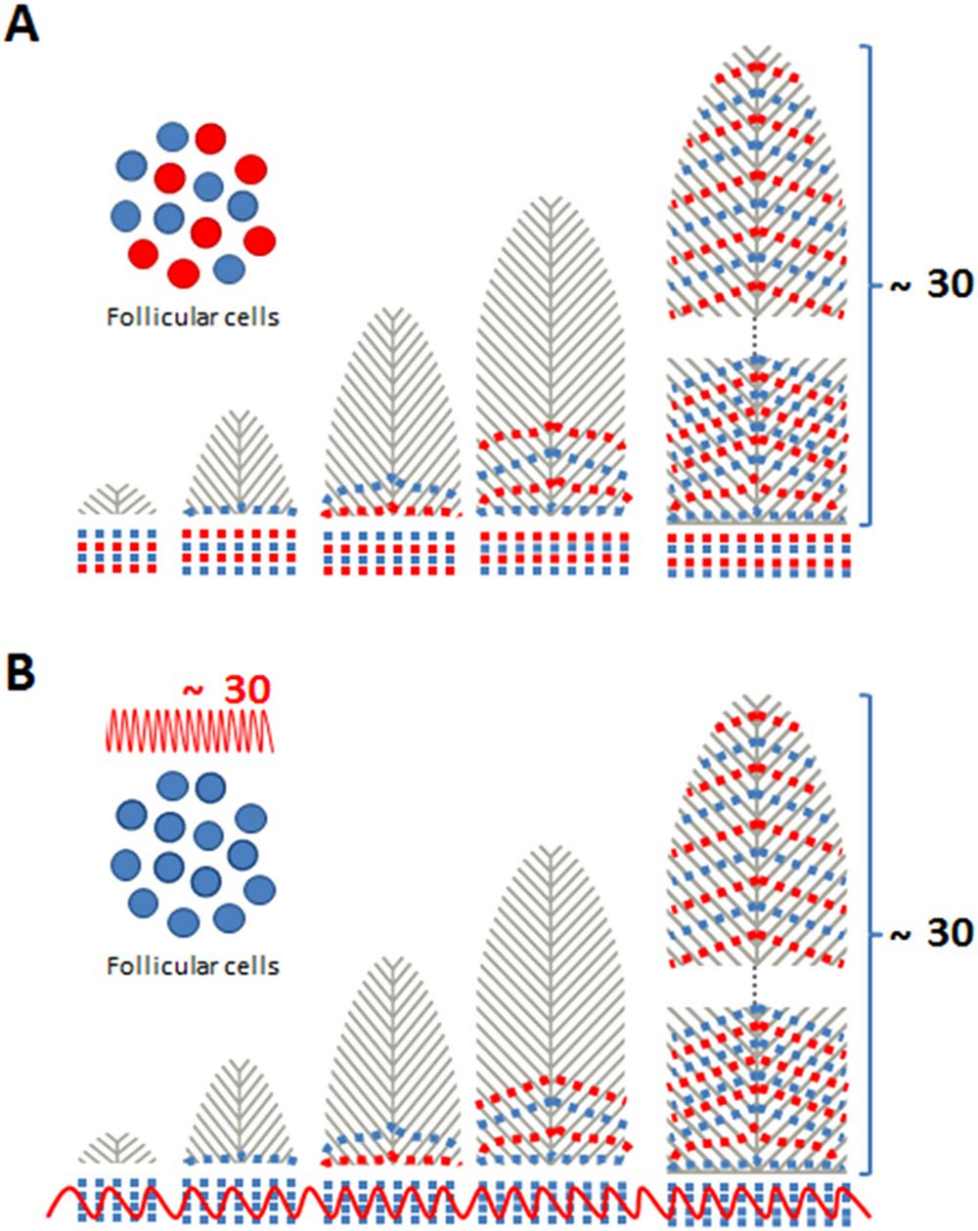
Models of pattern formation in feathers. (**A**) The observed Zn pattern formation (~30 regularly spaced deposits of Zn) may be the result of a pre-existing heterogeneity amongst feather stem cells with differential Zn uptake giving rise to a mosaic with a subsequent Zn pattern formation. (**B**) The absence of any ultrastructural differences (see Fig. 3) gives rise to a speculative, alternative explanation: The number of alternate lines of high and low Zn (~30) approximates the estimated overall period of growth, i.e. ~30 days. The Zn pattern formation might thus reflect diurnal oscillation in systemic Zn levels.

The elemental maps reveal patterns that appear to be independent from any detectable variations in pigmentation, density or macroscopically visible changes in the structure of the feather, providing evidence for previously unknown topobiological mechanisms of integumental tissue development^3, 4^. Pattern formation during feather growth may be the result of a pre-existing heterogeneity amongst stem cells or may reflect oscillations of systemic levels as the result of a physiological process, such as sleep/wake, feed cycle or periodic renal clearance (Fig. 2). Recently, using a similar technical approach, it has been shown that a previously undescribed strontium distribution in shark vertebrae correlates with the age of the individual and allows for age assessment to be conducted in species where visual assessment of growth bands was not possible^5^. These newly discovered growth patterns in feathers are expected to similarly have practical utility in the retrospective, time-stamped assessment of animal health and its relationship to environmental stressors^6^.

## Results

### X-ray fluorescence microscopy

#### Zinc

**Figure 3:**
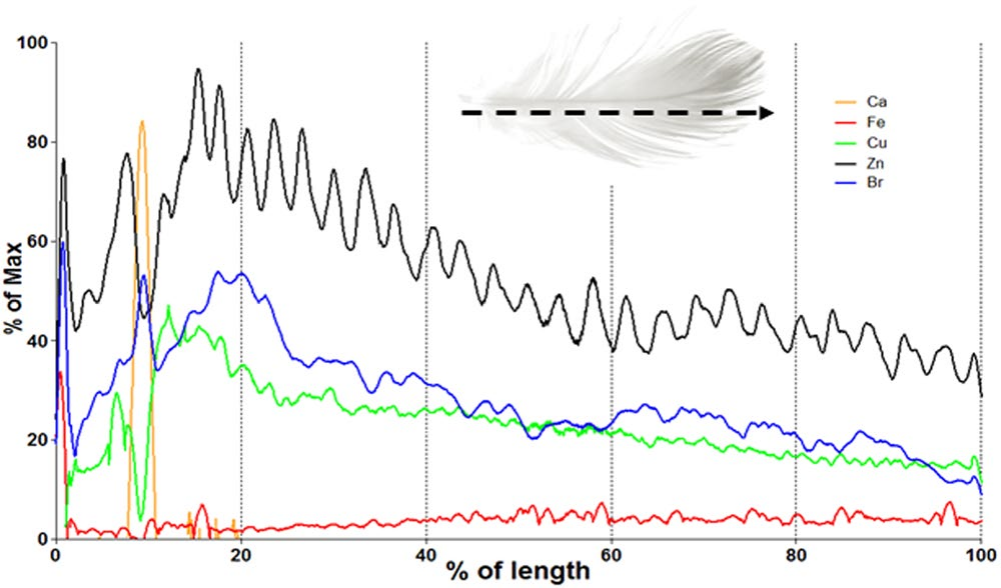
(**A**) A typical line profile of elemental distribution along the calamus and rachis of a shearwater breast feathers. The elements Ca, Fe, Cu, Zn and Br are found along the length of feather in discrete non-overlapping patterns, suggesting independent mechanisms of deposition. Data was plotted along the length of the rachis and calamus after normalising to the maximum intensity.

**Figure 4:**
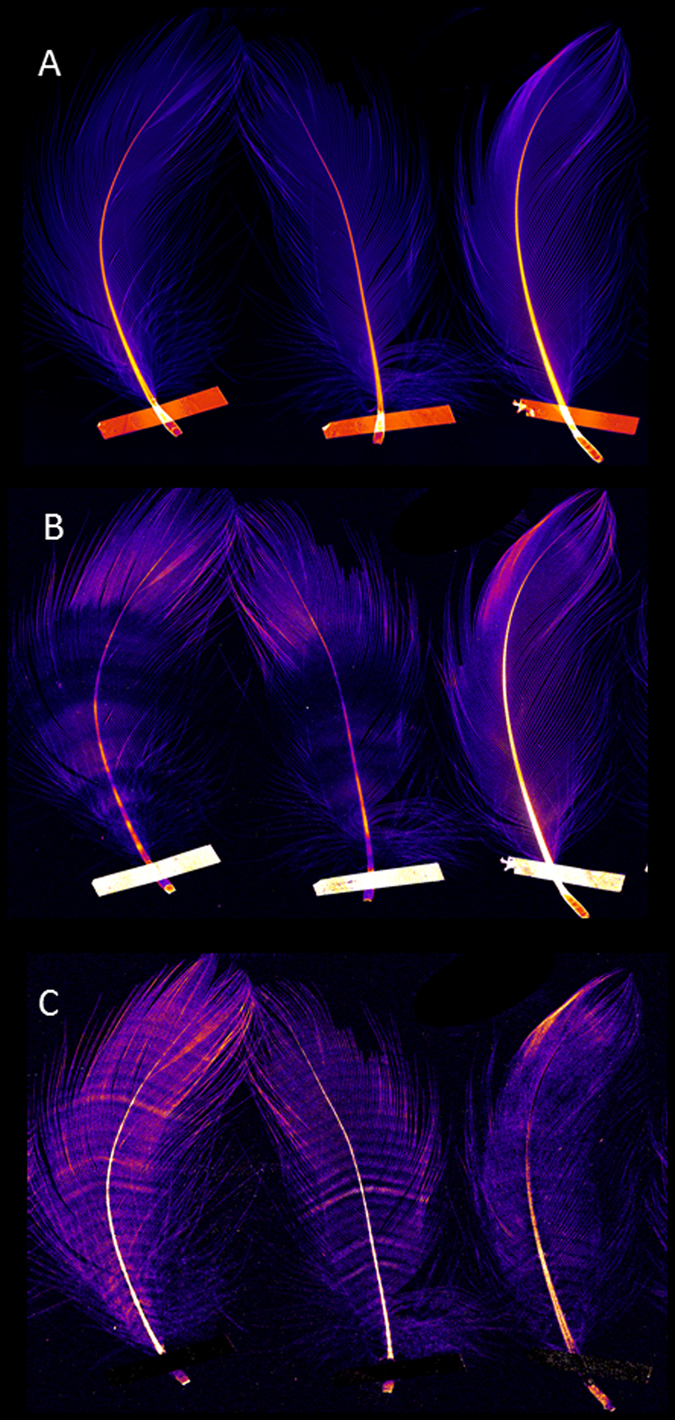
Compton (**A**) Br (**B**) and Zn (**C**) patterns Streaked Shearwater feathers (*Calonectris leucomelas*). Three breast feathers from three individual Streaked Shearwater, scanned simultaneously in high resolution, demonstrate the distinct banding of the Br distribution and regular patternation of Zn along the length of the rachis. Maps of Compton inelastic scattering were constructed from the XFM to inform the anatomy and composition of each sample. The intensity of Compton scatter is equivalent to the mass and density of material encountered by the incident beam and was used to correlate changes in elemental concentration and distribution with potential changes in the composition of each sample. The deposition pattern of the elements Br and Zn is independent of regional mass and density in the feathers.

Zn was detected in all structures of the feather, i.e. calamus, rachis and vanes (Figs 1 and 3). The concentration distribution of Zn along the rachis and vanes revealed a distinct and repetitious banding pattern, akin but unrelated to growth bands^7, 8^, with concentration peaks occurring approximately 30 times, every 1–1.5 mm along the rachis and radiating out into the vanes. The Zn distribution pattern, as was the case with the other elements described below, did not co-vary with tissue density or compositional changes in the feathers as measured by Compton scattering and small angle X-ray scattering (Figs 3 and 4).

**Figure 5:**
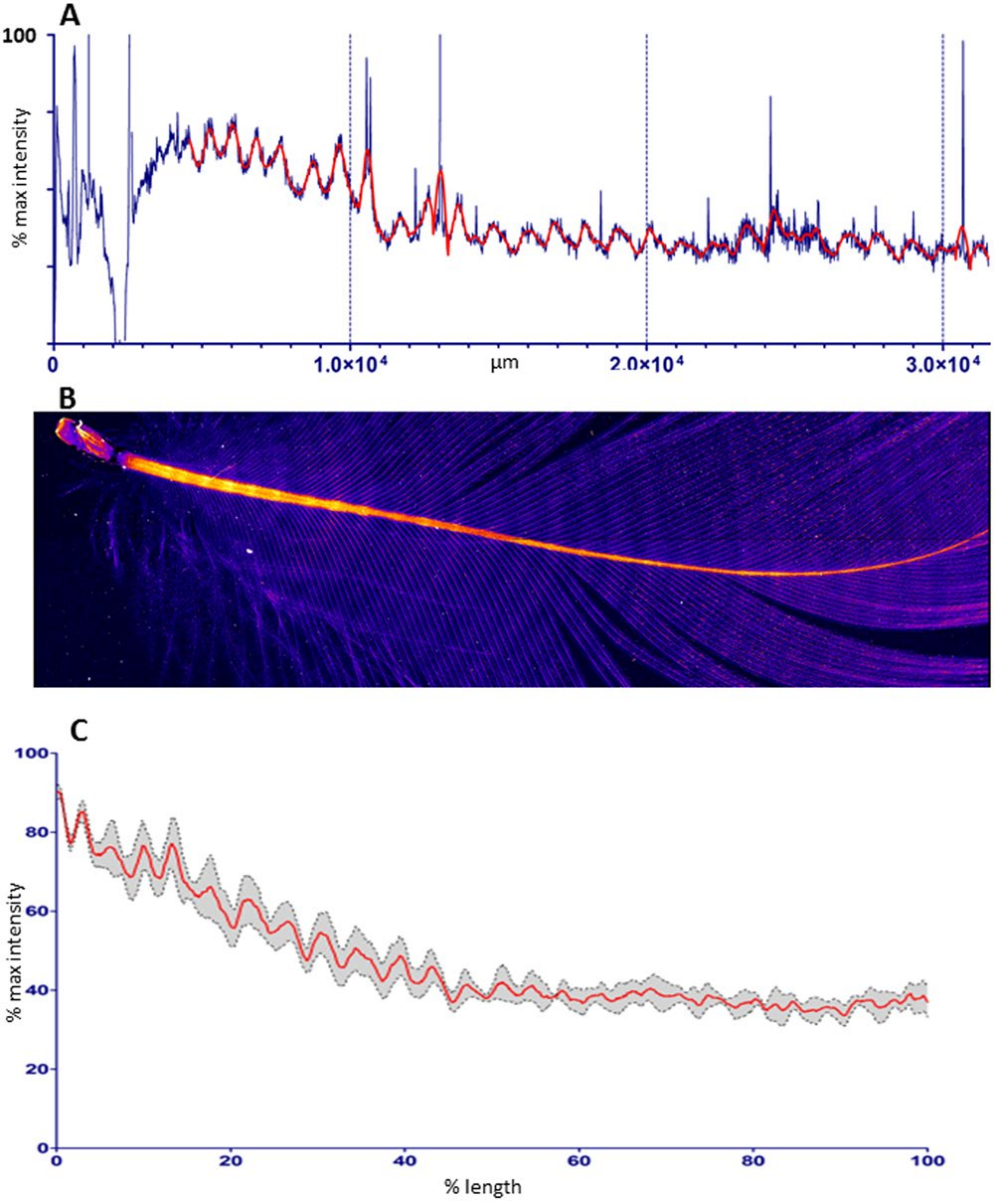
Zn distribution within a Flesh-footed Shearwater beast feather. The line profile (**A**) derived from the Zn map shown in (**B**) illustrates the inter-individually consistent periodicity in elemental Zn distribution. The red line shows the observed pattern after smoothing to reduce noise. The mean of Zn distributions is shown (**C**). The individual Zn distributions were taken along the rachis of each sample, normalised to length and intensity then used to create an average distribution across feather from multiple birds (n = 4) demonstrating that the number of Zn bands is consistently 30–32.

**Figure 6:**
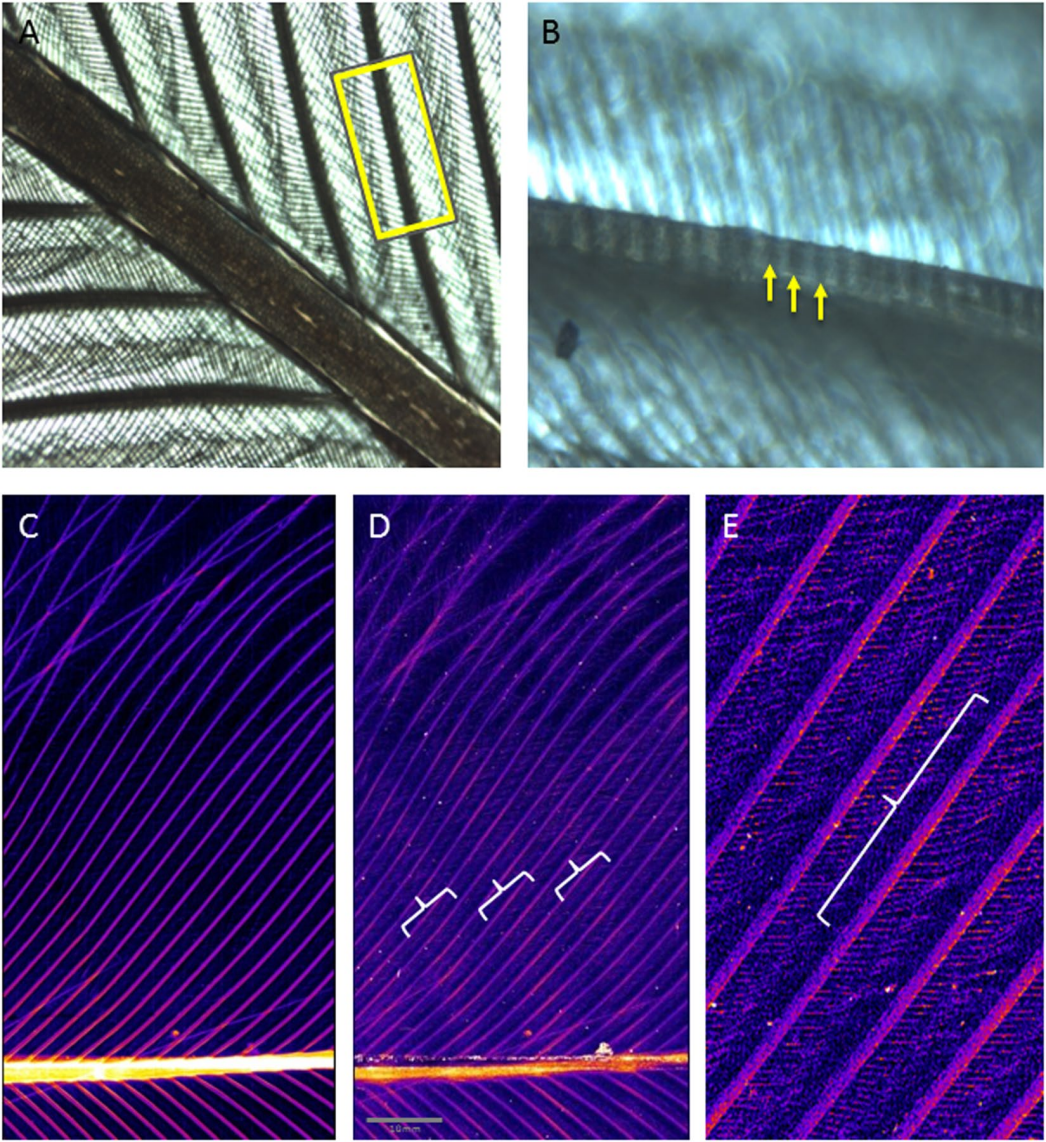
Examples of other visible periodic elements of a feather (**A**,**B**) with the invisible elemental periodicity observable within Zn distribution (**C**–**E**). Other examples of periodicity in feathers include the barbs and barbules themselves but also fault bars, alterations in the structure of the interlocking barbules caused by malnutrition or pathology, and growth bars, periodic pigment changes which often correlate diurnally with the time of feather growth. The periodicity observable in the Zn distribution does not correlate with any visible abnormalities, pigment changes, structural alterations or anatomy.

While the concentrations of Zn in or between the deposition bands, as well as the overall concentration in the feathers may be variable, the regularly banded pattern always remained clearly discernible. The average Zn distribution pattern in feathers sampled from different birds is shown Fig. 5. This pattern was consistently observed across the different species of Flesh-footed, Streaked and Short-tailed Shearwaters and did not correlate with any other visible repeating structures (Fig. 6).

#### Calcium

Across species, calcium (Ca) showed distributions (Figs 1 and 3) with the highest concentrations consistently found at the calamus to skin interface. This local calcium deposition appeared to co-locate with higher concentration in copper, zinc and bromine. Otherwise, only a low and varying concentration of calcium was found across the entire the feather structure.

#### Bromine

**Figure 7:**
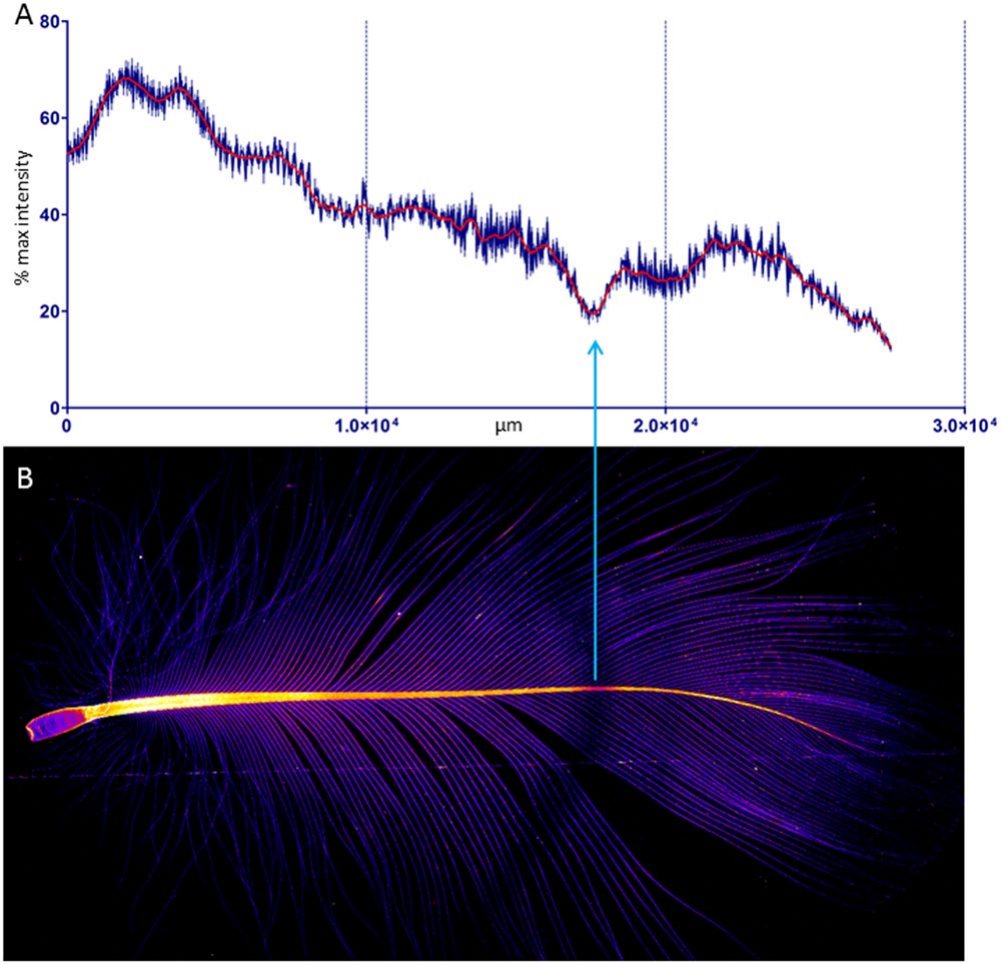
Br distribution within a Flesh-footed Shearwater breast feather. Image and line profile of a Flesh-footed Shearwater breast feather demonstrating a typical low concentration banding anomaly observed in in shearwater breast feathers. In contrast to Zn, the Br free banding is an inconsistent feature that does not correlate with any of the other distributions measured.

Bromine (Br) was ubiquitously and prominently observed in the calamus, rachis and vanes (Figs 1, 3, 4 and 7). While there were frequently discernible bands of low concentration of bromine, their pattern, unlike zinc, was irregular and varied between feathers (Figs 3 and 7). These alternate bands of low and high Br concentration did not co-locate with any of the other elements analysed. The typical fan shaped of geometry of the Br bands is consistent with the known helical displacement and deletion of keratinocyte during feather growth^9^. It remains to be established as to whether the Br pattern, which is much less regular than the Zn pattern, reflects of changes in systemic Br concentration, possibly feeding dependent, or functional heterogeneity between the stem cells in the feather follicle.

#### Copper

Copper (Cu), together with zinc, iron and manganese, is regularly found in feather pigments^10, 11^ and necessary for a variety of physiological functions^12, 13^. All species analysed showed a mostly uniform distribution along the rachis with somewhat lower concentrations in the calamus and vanes (Fig. 1). Concentrations along the rachis were variable without any regular patterns or visible banding (Fig. 3).

#### Iron

Iron (Fe), important in many catalytic processes and found complexed with melanin in various feather pigments^10, 14^, was mainly seen in the feather vanes while mostly absent from the rachis (Fig. 1). Random, high concentration patches of Fe frequently observed on the calamus, were due to external contamination, found to be either strongly adherent soil particles, that could not be washed off or small remnants of tissue or blood, usually close to the root of the plucked feathers. The concentration of Fe was higher towards the distal end of the feather which is the inverse to the all the other elements observed (Fig. 3).

## Discussion and Conclusion

Synchrotron radiation X-ray fluorescence has previously been shown to be useful for the study of feather barb composition^15^. The Australian Synchrotron scanning X-ray fluorescence microprobe used in this study deploys a 16 keV incident energy beam focussed to several microns to create images with pixel sizes between 5–70 μm. It thus allows the acquisition of elemental concentration maps of whole feathers of previously unreached sensitivity and resolution for a wide range of elements with an atomic number greater than 19 (potassium). Our data demonstrate that the atomic elements Zn, Ca, Br, Cu and Fe are non-uniformly distributed throughout the length of a feather. This uneven distribution of elements suggests that for accurate estimations of the elemental content of a feather requires the measurement of whole feathers rather than the frequently practised sub-sampling of feathers, such as clippings from the tip of a feather^2, 16, 17^.

Apart from the practical utility of our observations for studies that use seabirds as bio-indicators of environmental health^17–19^, we demonstrate the presence of previously unseen, distinct spatial distribution pattern for some chemical elements, such as Zn, that contain in itself potentially important topobiological information not contained in or obviously related to other features, such as visible feather pigmentation or known growth bands^20^.

Zinc (Zn) is an essential trace element for growth, enzyme structure and function, immune function and proper skeletal development and maintenance^21–24^. The pattern of the observed Zn bands, in all samples and across species, as they radiate out through the vanes is consistent with the proximal helical displacement of keratinocytes that result in the formation of barbs^25^. The Zn banding pattern was found in all shearwater species investigated and was also observed in *Diomedea exulans* (Wandering Albatross) (Suppl. 1) and thus seems to have evolved within the larger Procellariiformes order. Our data obtained from Short-tailed shearwater demonstrated that the patterned Zn deposition is not confined to breast feathers but is also present in all the major pteryla (Suppl. 2). Zinc distribution data obtained from the feathers of other, evolutionary significantly more distant species, including *Dromaius novaehollandiae* (Emu), *Apteryx* sp. (Brown Kiwi), *Pelagodroma marina* (White-faced Storm Petrel), *Stagonopleura guttata* (Diamond Firetailed Finch), *Gallus gallus* (Red Junglefowl), *Anas platyrhynchos* (Mallard) and *Cygnus atratus* (Black Swan) (Suppl. 1)^26^ did not reveal the presence of a regular Zn banding pattern. It raises the prospect that the study of hidden elemental distribution patterns, unrelated to the visible and behaviourally salient pigmentation of bird feathers, may be useful in the clarification of some evolutionary lineages and relationships^27^.

While the underlying physiological mechanism that gives rise to the highly regular and across Procellariiformes species consistent Zn banding pattern remains to be elucidated, the number of discrete bands equates to the known number of days of active feather growth (approx. 30) and thus suggests a regular diurnal pattern of Zn deposition. More precise knowledge on the interspecies variability of the growth and moult of particularly Shearwater feathers is required in order to assess the relevance of moult strategies and diet on elemental deposition^28^. Due to its regularity, the Zn banding may be suitable for ptilochronological analysis^8^. We speculate that such time-dependant periodicity related to a vital trace element has the potential to inform retrospectively, similarly to anomalies in tree rings, about disruptions in the normal ptilochronology of feather growth and thus be a useful marker of past exposure to stressors affecting the health status of the bird.

Characteristic, albeit less regular, patterns were also identified for Br. The bands of Br deposition throughout the feather were of variable width with spacing distances equivalent to 1 to 3 Zn bands. The spacing pattern of Br did not correlate with any discernible variations in the underlying molecular structure of the feather. Whether the bands of reduced Br deposition relate to periodic increases and decreases of Br from marine dietary sources remains to be elucidated.

Feathers are often used as indicators of individual, population and environmental health^19, 29^. High definition elemental imaging the of bird feathers using the advanced X-ray Fluorescence Microscopy beam-line at the Australian Synchrotron allowed us to describe, in detail, previously undocumented properties of the feather, notably distribution patterns of vital chemical elements that appear to be deposited independently from pigmentation or structural variations in the feathers.

High resolution elemental analysis of bird feathers thus promises new insights into physiological regulation during the period of feather growth, including the deposition of contaminants during the growth period as well as more subtle evidence for the impact of stressors when readily detectable anomalies cannot be found. Using high resolution spatial mapping of elemental distributions by synchrotron X-ray microscopy in hard biological structures, we have demonstrated its significant potential in the field of developmental biology through its ability to retrospectively inform on physiological variations or developmental stem cell differentiation, such as the description of previously unknown age-dependent strontium distributions in shark vertebrae that can enable accurate age assessment in a species in which this had not previously been possible^5^. More detailed studies are required to ascertain whether the observed variations in elemental depositions are related to variations in the feather keratin structure.

Although this study utilises synchrotron radiation as the primary x-ray source, recent advances in source anodes allow comparable X-rays to be produced by smaller laboratory instruments^30^. Techniques, such as used for this study, are thus set to become more widely available increasing the range and common use of X-ray microscopy and diffraction techniques.

## Materials and Methods

### Sampling

Breast feathers from Flesh-footed shearwater fledglings (n = 10 birds, 4–8 feathers each, approx. 80 days old) used for this study were collected on Lord Howe Island, Australia (32.53**°**S, 159.08**°**E,) during late-April to early-May 2011. Streaked Shearwater breast feathers (n = 8 birds, 4–8 feathers each) were obtained from fledglings on Mikura Island (33°52′N, 139°14′E, Izu Islands, Japan) (mid-November) and from fresh adult carcasses during chick rearing on Awa Island (38°27′N, 139°13′E, Niigata, Japan) (Aug-Oct 2011) during the 2011 breeding season. Four to six adult breast feathers were collected from Mikura and Birou (31°25′N, 131°7′E, Miyazaki, Japan) Islands during the incubation period (mid-July) in the 2012 breeding season. At Mikura Island additional breast feathers were also obtained from fresh carcasses. Short-tailed shearwater feather samples (n = 4 birds) were taken from carcases collected (n = 44 birds) from Bundeena Beach (34°04′S, 151°09′E, Sydney, Australia) in September 2014. Samples were collected with the permission of the Lord Howe Island Board and Ministry of the Environment, Government of Japan, under the approval of James Cook University and University of Tasmania Animal Ethics Committees. All procedures were performed in accordance with relevant guidelines and regulations.

All feathers were placed in sterile polyethylene bags and stored at −20 °C prior to analysis.

### X-ray Fluorescence Microscopy

Whole breast feathers were sandwiched between two layers of 3.6 µm Mylar film and then fixed within a Perspex frame sample holder with adhesive Kapton tape (DuPont). The sample holder was mounted onto a high precision positioning stage. An image captured by a high resolution CCD mounted behind the stage was used to determine the coordinates of the scan areas. A 16 keV incident energy beam was focussed to 2–3 µm through a Kirkpatrick-Baez mirror system. The sample was scanned through this beam and the resulting fluorescence spectra were collected using the Maia 384 large solid-angle detector applying the dynamic analysis method, described by Ryan *et al*.^31^. The data was collected and analysed using GeoPIXE (http://www.nmp.csiro.au/GeoPIXE.html).

## Electronic supplementary material

Supplementary information
