# Supplementary material for: The Topobiology of Chemical Elements in Seabird Feathers
Source: Sci Rep. 2017 May 17;7:1998. doi: 10.1038/s41598-017-01878-y (PMC5435718; doi:10.1038/s41598-017-01878-y)

The Topobiology of Chemical Elements in Seabird Feathers

Nicholas Howell[a], Jennifer L. Lavers[b], Sayaka Uematsu[c,d], David Paterson[e], Daryl L. Howard[e], Kathryn Spiers[e], Martin D. de Jonge[e], Tracey Hanley[a.], Richard Garrett[a,e], Richard B. Banati[a,f]

[a.] Australian Nuclear Science and Technology Organisation (ANSTO), Lucas Heights, Australia

[b.] Institute for Marine & Antarctic Studies, University of Tasmania, Australia

[c.] James Cook University, Cairns, Australia

[d.] NRDA Asia, Tokyo, Japan

[e.] Australian Synchrotron, Melbourne, Australia

[f.] National Imaging Facility at Brain and Mind Centre (BMC), Faculty of Health Sciences, University of Sydney

Authors for correspondence

Nicholas Howell and Prof. Richard B. Banati
Locked Bag 2001 Kirrawee DC NSW 2232 Australia, +61 2 9717 3071,
nicholas.howell@ansto.gov.au; richard.banati@ansto.gov.au

Dr. Jennifer L. Lavers
Institute for Marine and Antarctic Studies, University of Tasmania, 20 Castray Esplanade, Battery Point, Tasmania, 7004, Australia
jennifer.lavers@utas.edu.au

Supplementary Figure 1.
Comparison of Zn distribution across species

(A) Black Swan, (B) Kiwi, (C) Storm Petrel, (D) Emu, (E) Firetail Finch, (F) Chicken, (G) Mallard, (H) Wandering Albatross

(I) Phylogenetic relationship of the species measured so far indicates that the periodic distribution of Zn is restricted to order Procellariiformes

Supplementary Figure 2.
Feathers of the major pteryla of Short-tailed Shearwater .

Regular patternation of Zn distribution observable from feathers of major pteryla of the Short-tailed Shearwater (A) capital tracts, (B) primary remiges, (C) secondary remiges, (D) rectrices, (E) femoral tract, (F) dorsopelvic tract, (G) interscapular tract, (H) pectoral tract.

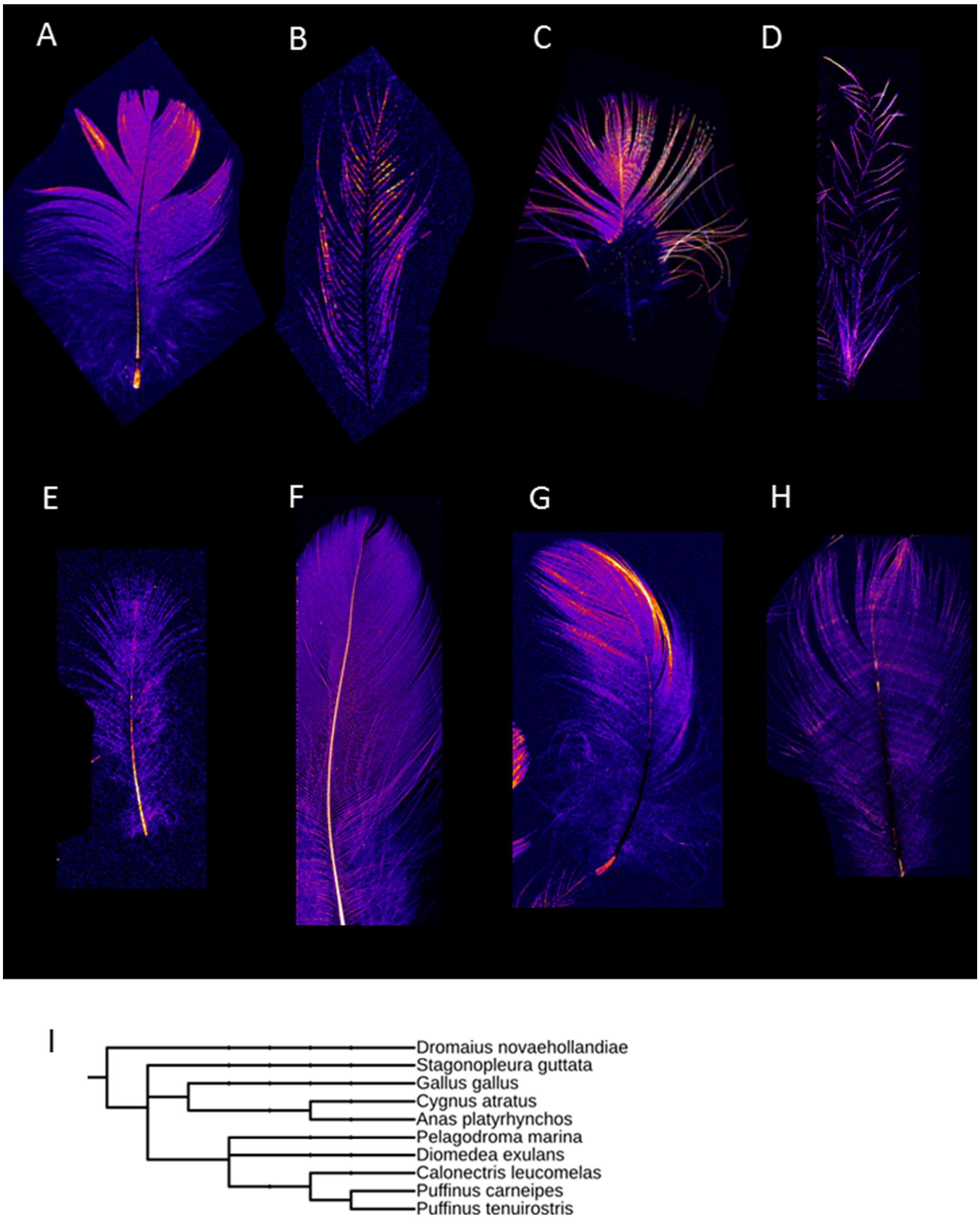
A
B
C
D
E
F
G
H
I
Dromaius novaehollandiae
Stagonopleura guttata
Gallus gallus
Cygnus atratus
Anas platyrhynchos
Pelagodroma marina
Diomedea exulans
Calonectris leucomelas
Puffinus carneipes
Puffinus tenuirostris

Supplementary Figure 2.

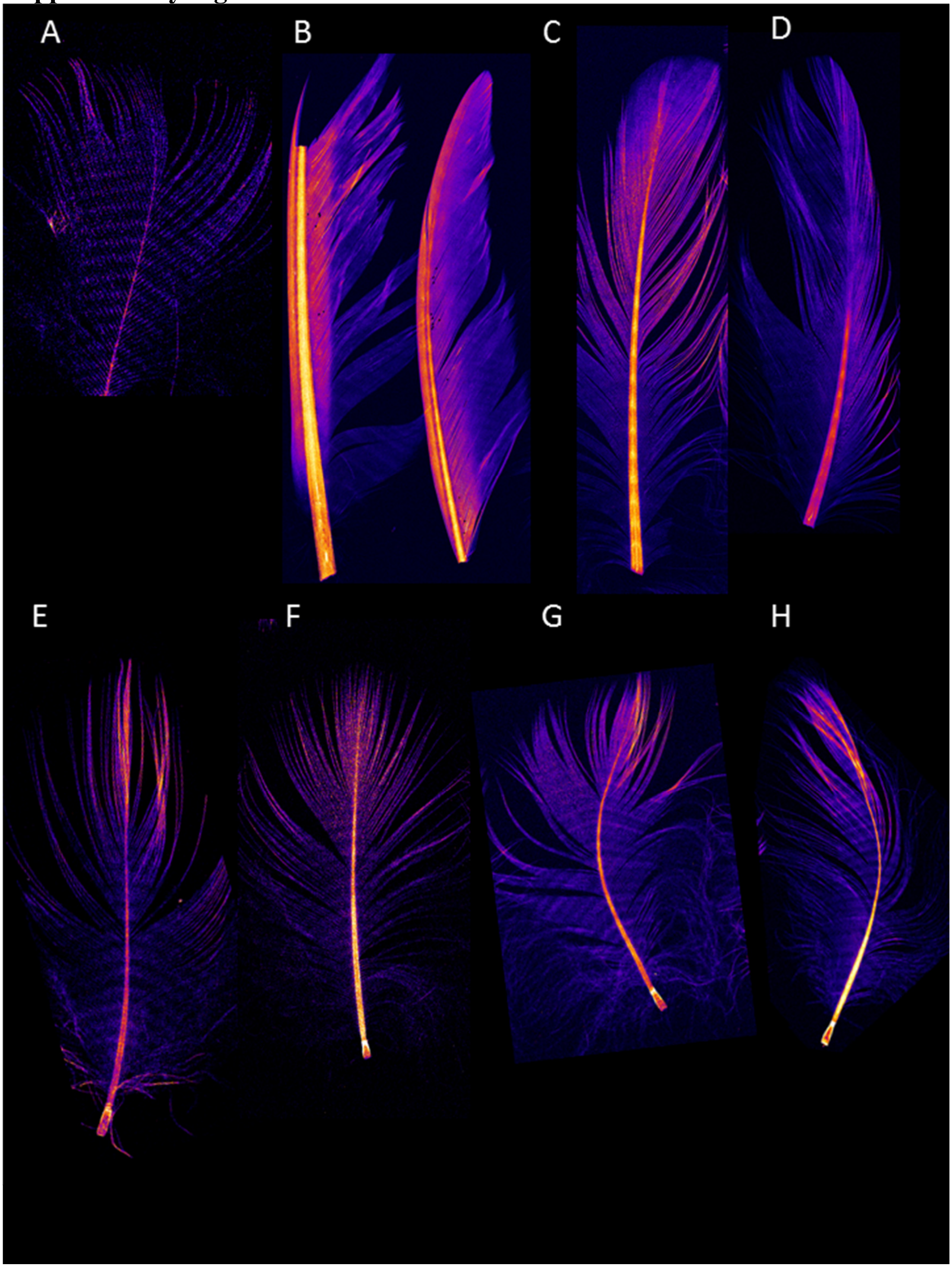

Supplement: Supplementary file 1 — Supplementary information [file 41598_2017_1878_MOESM1_ESM.pdf]
